# Chemosensitization of *Fusarium graminearum* to Chemical Fungicides Using Cyclic Lipopeptides Produced by *Bacillus amyloliquefaciens* Strain JCK-12

**DOI:** 10.3389/fpls.2017.02010

**Published:** 2017-11-27

**Authors:** Kihyun Kim, Yoonji Lee, Areum Ha, Ji-In Kim, Ae Ran Park, Nan Hee Yu, Hokyoung Son, Gyung Ja Choi, Hae Woong Park, Chul Won Lee, Theresa Lee, Yin-Won Lee, Jin-Cheol Kim

**Affiliations:** ^1^Department of Agricultural Chemistry, Institute of Environmentally-Friendly Agriculture, College of Agriculture and Life Sciences, Chonnam National University, Gwangju, South Korea; ^2^Department of Agricultural Biotechnology, Seoul National University, Seoul, South Korea; ^3^Center for Eco-Friendly New Materials, Korea Research Institute of Chemical Technology, Daejeon, South Korea; ^4^World Institute of Kimchi, Korea Food Research Institute, Gwangju, South Korea; ^5^Department of Chemistry, College of Natural Sciences, Chonnam National University, Gwangju, South Korea; ^6^Microbial Safety Team, Department of Agro-Food Safety and Crop Protection, National Institute of Agricultural Sciences, Wanju, South Korea

**Keywords:** *Bacillus amyloliquefaciens* JCK-12, Fusarium head blight, cyclic lipopeptides, synergistic antifungal effect, chemosensitization

## Abstract

Fusarium head blight (FHB) caused by infection with *Fusarium graminearum* leads to enormous losses to crop growers, and may contaminate grains with a number of Fusarium mycotoxins that pose serious risks to human and animal health. Antagonistic bacteria that are used to prevent FHB offer attractive alternatives or supplements to synthetic fungicides for controlling FHB without the negative effects of chemical management. Out of 500 bacterial strains isolated from soil, *Bacillus amyloliquefaciens* JCK-12 showed strong antifungal activity and was considered a potential source for control strategies to reduce FHB. *B. amyloliquefaciens* JCK-12 produces several cyclic lipopeptides (CLPs) including iturin A, fengycin, and surfactin. Iturin A inhibits spore germination of *F. graminearum.* Fengycin or surfactin alone did not display any inhibitory activity against spore germination at concentrations less than 30 μg/ml, but a mixture of iturin A, fengycin, and surfactin showed a remarkable synergistic inhibitory effect on *F. graminearum* spore germination. The fermentation broth and formulation of *B. amyloliquefaciens* JCK-12 strain reduced the disease incidence of FHB in wheat. Furthermore, co-application of *B. amyloliquefaciens* JCK-12 and chemical fungicides resulted in synergistic *in vitro* antifungal effects and significant disease control efficacy against FHB under greenhouse and field conditions, suggesting that *B. amyloliquefaciens* JCK-12 has a strong chemosensitizing effect. The synergistic antifungal effect of *B. amyloliquefaciens* JCK-12 and chemical fungicides in combination may result from the cell wall damage and altered cell membrane permeability in the phytopathogenic fungi caused by the CLP mixtures and subsequent increased sensitivity of *F. graminearum* to fungicides. In addition, *B. amyloliquefaciens* JCK-12 showed the potential to reduce trichothecenes mycotoxin production. The results of this study indicate that *B. amyloliquefaciens* JCK-12 could be used as an available biocontrol agent or as a chemosensitizer to chemical fungicides for controlling FHB disease and as a strategy for preventing the contamination of harvested crops with mycotoxins.

## Introduction

*Fusarium graminearum* causes Fusarium head blight (FHB), a globally devastating fungal disease of small grain cereals, especially on wheat and barley (McMullen et al., 1997). FHB results in extensive yield loss of grains (Goswami and Kistler, 2004). Over the past several decades, severe FHB outbreaks have occurred in wheat growing areas across the world, and an estimated US$ 8 billion loss was incurred from FHB in the United States between 1993 and 2001 (Nganje et al., 2004). Moreover, due to climate change, it is expected that FHB epidemics will become more severe and further losses in crop yield will occur (Madgwick et al., 2011). FHB also reduces the quality and feeding value of crops by producing various toxic metabolites, including trichothecenes and zearalenone mycotoxins. Deoxynivalenol (DON), the most important trichothecene, poses a significant threat to animal health and food safety (Marasas et al., 1984; Joffe, 1986; Snijders, 1990; McMullen et al., 2012) and facilitates disease development by acting as a virulence factor (Desjardins et al., 1996).

Fusarium head blight disease can be prevented or controlled through several strategies including crop rotation, tillage practices, fungicides application, and planting less susceptible cultivars. Among these, chemical control applied during the anthesis period is the most effective strategy (Homdork et al., 2000; Mesterházy et al., 2011). However, because of the rising cost of chemical pesticides, growing perception about their negative effects such as accumulation of toxic residues in crops and development of resistance in pathogens in recent years, it is urgently required to develop safer control agents fulfilling the consumer demand of pesticide-free production. Moreover, FHB management using chemical fungicides has a major problem, in that *F. graminearum* infections can occur beyond the 30-day preharvest interval when chemical fungicides cannot be legally applied (Francl et al., 1999; McMullen et al., 2012). These late-season infections lead to significant accumulation of the *Fusarium*-produced mycotoxin DON. Thus, biological control using antagonistic microorganisms has been suggested as an alternative strategy and can be used as part of an integrated management for FHB disease (Dal Bello et al., 2002; Kim et al., 2003).

Several bacterial strains have been reported as antagonistic microorganisms against *F. graminearum* (Zhao et al., 2014). *Bacillus* strains have especially been spotlighted as effective biological control agents due to their strong antimicrobial activities as well as their resistance to environmental stress conditions (Nagórska et al., 2007). The biocontrol mechanisms of *Bacillus* strains are related to antagonism, competition, systemic resistance induction, root colonization, and promotion of plant growth (Ongena and Jacques, 2008). In particular, some *Bacillus* strains produce a variety of lipopeptides with considerable structural diversity, which are key players in their antagonistic activities toward viruses, mycoplasmas, bacteria, yeast, fungi, and nematodes (Zeriouh et al., 2011). *B. subtilis* or related species have been known to produce three cyclic lipopeptides (CLPs), iturin A, fengycin, and/or surfactin. The iturin family shows a strong antifungal activity against both yeast and fungi, (Moyne et al., 2001; Yu et al., 2002), whereas the fengycin family has a broad range of fungitoxic activity, mainly against filamentous fungi, and are also known to elicit induced systemic resistance (ISR) (Vanittanakom et al., 1986). The surfactin family also induces ISR in plants and functions as strong biosurfactants (Ongena and Jacques, 2008). To date, the effectiveness of *Bacillus* strains for the control of *F. graminearum* has been mainly evaluated using bacterial culture supernatants, whereas few studies have been performed using a formulation based on *Bacillus* strains in field trials (Khan et al., 2004; Palazzini et al., 2007; Khan and Doohan, 2009). Moreover, no study has yet been conducted to investigate the synergistic effects of biological control agents and chemical fungicide mixtures under the field condition.

In the last decade, crop protection using chemical fungicides has been an influential tool for the control of various plant pathogenic fungi. However, by the extensive use of chemical fungicides, there is now a significant increase in the incidence of resistance in fungal pathogens to agricultural fungicides. Therefore, chemical fungicides undermine the reliability of their effectiveness, which is relatively short-lived and ultimately uneconomical. Chemosensitization is one of the strategies to resolve this problem. It is based on the enhanced sensitivity of pathogen to fungicides by co-application with a non- or slightly active fungicidal substance and a commercial fungicide at levels where, neither compound alone would be effective. Since antifungal chemosensitization is considered a novel antifungal intervention strategy, the model fungal system has been used for the development of chemosensitizers to improve the efficacy of conventional agents against fungal pathogens (Niimi et al., 2004; Kim et al., 2008).

In this study, we isolated *B. amyloliquefaciens* strain JCK-12 possessing strong antifungal activity against *F. graminearum* and observed that *B. amyloliquefaciens* JCK-12 and several chemical fungicides in combination have synergistic antifungal interactions. Our study shows the possibility of *B. amyloliquefaciens* JCK-12 as an available biocontrol agent for controlling FHB or as a chemosensitizer to improve the sensitivity of chemical fungicides. Therefore, the objectives of this study were (1) to identify useful bacterial strains for FHB control, (2) to determine the antagonistic efficiency of the JCK-12 strain for FHB control, and (3) to increase the utilization value of JCK-12 by characterizing its biocontrol mechanism.

## Materials and Methods

### Strains, Culture, and Growth Conditions

The *F. graminearum* wild-type strain Z-3639 (Bowden and Leslie, 1999), hH1-GFP (Hong et al., 2010), and HK12, a constitutive GFP expresser (Son et al., 2011b), was grown on potato dextrose agar (PDA) at 25°C for propagation of mycelium and in carboxyl methyl cellulose (CMC) medium for asexual sporulation (Cappellini and Peterson, 1965). For evaluating antifungal activity, *Botrytis cinerea, Colletotrichum coccodes, Fusarium oxysporum* f. sp. *niveum, Phytophthora capsici*, and *Rhizoctonia solani* were received from Dr. GJ Choi, Korea Research Institute of Chemical Technology, Daejeon, Korea. *Fusarium oxysporum* f. sp. *lycopersici* (KACC40043), *Fusarium verticillioides* (KACC45825), and *Magnaporthe oryzae* (KACC46522) were received from the Korean Agricultural Culture Collection (Wanju, Korea). We also received *Raffaelea quercus-mongolicae* from the National Institute of Forest Research (Seoul, South Korea). Each plant pathogenic fungus was incubated on PDA except for *P. capsici*, for which V8 agar medium was used (Ristaino, 1990). Bacterial strains were inoculated in tryptic soy broth (TSB, Difco, Detroit, MI, United States) for 3 days at 30°C with agitation (200 rpm). All strains were stored in 20% glycerol at -70°C.

### Isolation of Rhizospheric Bacteria

Soil samples were taken from the fields grown tomatoes, peppers, or onions of four regions in Korea (Daejeon, Gwangju, Jeongeup, and Geoje). Samples were collected from soil which around plants roots between 10 and 20 cm depth, placed in a cool box for transport, and stored at 4°C. Each 1 g of soil sample was suspended in 10 ml of sterile distilled water and shaken vigorously for 2 min. The supernatant was serially diluted in sterile distilled water (10^-1^ to 10^-7^), and plated on tryptic soy agar medium (TSA, Difco). After incubation at 30°C for 1–2 days, isolated colonies were purified on TSA, and stored cryogenically at -70°C.

### Screening of Antifungal Bacteria by a Dual Culture Bioassay

The antagonistic activity of bacterial strains isolated from rhizospheres against *F. graminearum* was evaluated by a dual culture bioassay (Lam et al., 2000). A mycelial plug (5 mm diameter) of *F. graminearum* was placed at the center of the PDA plate and the bacterial strains were inoculated at four cardinal points 25 mm from the center of the plate. As control, only *F. graminearum* was inoculated. After 4 days of incubation at 25°C, the inhibition zone was measured. Inhibition was graded by relating the inhibited growth area per inoculation streak to the total area of the Petri dish. The following scale was used: -, no visible inhibition; ^∗^, no fungal growth on 0.1–3% of plate area/bacterial streak; ^∗∗^, no fungal growth on 3–8% of plate area/bacterial streak; ^∗∗∗^, no fungal growth on >8% of the plate area/bacterial streak. All data were obtained from three replicates.

### Inhibition of Spore Germination

The fermentation broth of each bacterial strain was centrifuged at 7,000 rpm for 20 min and the supernatant was filtered through a membrane filter (0.2 μm, Advantec, Tokyo, Japan). The axenic fermentation broth was tested for inhibitory activity against conidial germination of *F. graminearum*. Conidia were obtained from *F. graminearum* cultures incubated in CMC for 4–7 days. The spore suspension was filtered through four layers of sterile cheesecloth to remove mycelia and then adjusted to 1.0 × 10^6^ conidia/ml using a hemocytometer (Marienfeld Superior, Lauda-Königshofen, Germany). The axenic fermentation broth was applied at a concentration of 20, 10, 5, and 2.5%, and autoclaved TSB was used as a negative control. The final volume of the spore suspension was adjusted to 200 μl in each well of a 48-well plate and cultured at 200 rpm and 25°C. A spore was considered to be germinated if the germ tube was longer than or equal to the greatest dimension of the swollen spore (Dantigny et al., 2006). Three replicates of 100 spores for each experiment were observed using a Zeiss Axio Imager A2 Microscope (Carl Zeiss, Oberkochen, Germany).

### Molecular Identification of JCK-12

Identification of the bacterial strain was performed by evaluating the 16S rRNA, gyrase subunit A (*gyrA*), and bacterial RecA (*recA*) gene sequences. The genomic DNA of the bacterial strain was purified using the Bacterial genomic DNA purification kit (ELPIS-Biotech, Daejeon, South Korea) according to the manufacturer’s recommendations. Next, 16S rRNA, *gyrA*, and *recA* genes were amplified by PCR using specific primer pair sets (Supplementary Table 1) and sequenced (Genotech Co., Daejeon, South Korea). The gene sequences of some related species were downloaded from the GenBank database and aligned using BioEdit version 5.0.9.1 (Hall, 1999). The phylogenetic trees were constructed by the neighbor-joining (NJ) method in MEGA version 6.0 with 1,000 bootstrap replicates (Tamura et al., 2013).

### Antifungal Activity of JCK-12 against Various Plant Pathogenic Fungi

The antifungal potential of strain JCK-12 was examined by a dual culture assay using several plant pathogenic fungi (Fokkema, 1978). A 5 mm agar plug of each plant pathogenic fungus was placed on one side of a Petri dish containing PDA medium for most of the test fungi except for *P. capsici*, for which V8 agar medium was used. JCK-12 was then streaked at a distance of 35 mm from the fungal pathogen agar plug on the same dish. These paired cultures were incubated at 25°C. Plates inoculated only with test pathogens served as controls. The mycelial growth of each fungus was measured for 3–13 days until the mycelia of the corresponding control fungus grew to 35 mm. Antagonistic activities were evaluated by subtracting the distance of the fungal growth radius of the control culture. The experiments were repeated three times with three replicates.

### Extraction and Purification of Antifungal Cyclic Lipopeptides (CLPs)

After incubation of JCK-12 at 30°C for 3 days with agitation (150 rpm), the fermentation broth (3 l) was centrifuged at 8,874 × *g* for 20 min at 4°C to remove the bacterial cells. The bacterial cell-free supernatant of JCK-12 was partitioned twice with equal volumes of *n*-butanol. The butanol layer was concentrated to dryness on a rotary evaporator and then tested for inhibitory activity against *F. graminearum* spore germination. The butanol extract highly inhibited spore germination. The butanol extract (10 g) was separated by silica gel column chromatography (70–230 mesh, 300 g, 4.2 cm i.d × 60 cm; Merck, Darmstadt, Germany) using chloroform-methanol-distilled water-acetic acid (14:6:1:0.021, v/v/v/v) to yield three fractions F1–F3. Among these three fractions, only F2 was active. The bioautographic method revealed that CLP is responsible for the inhibitory activity of F2 against *F. graminearum* spore germination. To refine the active cyclic lipopeptides (CLPs), F2 (2.72 g) was dissolved in water and precipitated by adjusting the pH to 2.0 with 6 mol/l HCl. The solution was then stored overnight at 4°C, the resulting precipitates were collected by centrifugation at 8,874 × *g* for 20 min, and then extracted twice with methanol. The refined F2 was further purified on an ODS column (200–400 mesh, 55 g, 3.2 cm i.d. × 20 cm, Sigma–Aldrich, St. Louis, MO, United States) using methanol-distilled water (3:7), methanol-distilled water (5:5), methanol-distilled water (7:3), and methanol (100%) as mobile phases, and the antifungal active fraction was collected. The antifungal active fraction (148.3 mg) was next separated through a Sephadex LH-20 column (50 g, 1 cm i.d. × 40 cm; Sigma–Aldrich) using methanol (100%) and the separated antifungal compound was finally purified by preparative TLC (Prep TLC; 20 × 20 cm, 0.5 mm, Merck). The prep TLC was developed in chloroform-methanol-distilled water-acetic acid (14:6:1:0.021, v/v/v/v) as a mobile phase. The antifungal compound was then scraped from the developed Prep TLC and eluted with methanol, yielding the final compound (25.2 mg).

To confirm the production of other CLPs produced by JCK-12, the butanol extract of the JCK-12 culture supernatant was separated by prep TLC using chloroform-methanol-distilled water-acetic acid (14:6:1:0.021, v/v/v/v). After developing and drying the prep TLC plate, it was sprayed with water. Two white colored regions (fraction 1 and fraction 2) were separately scraped from the prep TLC and then extracted with methanol.

### Identification of Compounds Isolated from JCK-12 by HPLC and ESI-MS/MS Analyses

The antifungal active compound and the two CLP fractions, fraction 1 and fraction 2, isolated from JCK-12 were analyzed by HPLC using the following conditions. A C_18_ column (Atlantis T3, 5 μm, OBD 19 × 250 mm; Waters, Wexford, Ireland) was used, and the antifungal compound and fraction 1 were detected at 230 nm, whereas fraction 2 was detected at 215 nm. The mobile phases were as follows: solvent A, water with 0.1% trifluoroacetic acid (TFA); and solvent B, acetonitrile with 0.1% TFA. The compound was eluted with a linear gradient of solvent A increasing from 10 to 100% at a flow rate of 1 ml/min for 60 min. Iturin A (Sigma–Aldrich), fengycin (Sigma–Aldrich), and surfactin (Sigma–Aldrich) were used as standard chemicals. The retention times and UV spectra of the three CLPs were compared with those of the standard chemicals. To clearly confirm the identities of the CLPs isolated by prep TLC, electrospray ionization tandem mass spectrometry (ESI-MS/MS) analysis was performed on a SYNAPT G2 time-of-flight (TOF) mass spectrometry system (Waters, Manchester, United Kingdom) at a mass range of *m/z* 100–2,000.

### Inhibitory Activity of Commercial CLPs against *F. graminearum* Spore Germination

In order to examine the inhibitory activity of three CLPs, iturin A, fengycin, and surfactin, which were produced by JCK-12, against conidial germination of *F. graminearum*, the three standard chemicals were treated alone or in combination at a concentration range of 2.5–60 μg/ml. Commercial surfactin was dissolved in dimethyl sulfoxide (DMSO) at a concentration of 10 mg/ml and then diluted with methanol at a concentration of 1 mg/ml. Commercial fengycin and iturin A were dissolved in methanol and ethanol, respectively, at a concentration of 1 mg/ml. Mixtures of the three CLPs (iturin A+fengycin+surfactin = I+F+S) were prepared at ratios of 1:1:1, 1:1:0, 0:1:1, and 1:0:1 (w/w/w) in order to examine the synergistic effects between the CLPs. The mixtures were applied at a concentration range of 5–60 μg/ml. Ethanol and methanol were used as negative controls at 6% and DMSO at 0.6%, the concentration of which was determined through a preliminary experiment. The solvents at the given conditions did not affect the *F. graminearum* spore germination. The experiment was performed twice with three replicates and one hundred spores for each experiment were observed under the microscopy.

### Antifungal Activity of JCK-12

The antifungal activities of the JCK-12 culture supernatant and its butanol extract including three CLPs on the radial growth of *F. graminearum* were tested by a dual culture assay and pour plate method. For the dual culture assay, agar plugs (5 mm diameter) of actively growing mycelia were inoculated near the edge of a 50 mm-diameter Petri plate containing complete medium (CM) and incubated at 25°C. After 24 h, sterile paper disks with 10 μl of Luria-Bertani (LB) medium containing JCK-12 (1 × 10^7^ cfu/ml) or 10 μl of the butanol extract (60 μg/ml) were placed on the opposite edges of the Petri dishes and incubated at 25°C for 4 days. For pour plating, CM supplemented with the butanol extract (60 μg/ml) was used. These experiments were performed twice with three replicates.

To investigate the effect of the butanol extract on the mycelial morphology of *F. graminearum*, the fungus was cultured in liquid CM containing 0, 30, or 60 μg/ml of the butanol extract for 24 h at 25°C on a rotary shaker (200 rpm). Changes in hyphal morphology were observed with differential interference contrast (DIC) microscopy. DIC images were visualized using a DE/Axio Imager A1 microscope (Carl Zeiss, Oberkochen, Germany). To visualize green fluorescent protein (GFP) expression in cells, the 38H (excitation 470/40; emission 525/50) filter set was used.

To verify whether the effect of the butanol extract on mycelial morphology was derived from the antifungal action of the three CLPs produced by JCK-12, the fungus was cultured in liquid CM containing 15 μg/ml of commercial iturin A, or 1.25 μg/ml of a mixture of the three commercial CLPs for 24 h at 25°C on a rotary shaker (200 rpm). Changes in hyphal morphology were observed by DIC microscopy.

### Detection of Cell Membrane Permeability

The effect of butanol extract on cell membrane permeability was examined by propidium iodide (PI) staining as described in a previous study with slight modifications (Gao et al., 2016). Briefly, fungal strains grown for 24 h in 50 ml of liquid CM were incubated for an additional 24 h in CM supplemented with 30 μg/ml of the butanol extract. Mycelia were then harvested and incubated in 2 μM of PI for 20 min. To visualize the red fluorescent dye, a microscope using the filter set 15 (excitation 546/12; emission 590) was used. The experiments were repeated at least twice independently.

### Synergistic Effects of JCK-12 with Other Antifungal Compounds

To visualize the synergistic antifungal effect of the butanol extract including three CLPs and other antifungal compounds on the hyphal growth of *F. graminearum*, the fungus was grown on CM supplemented CLPs alone (10 or 30 μg/ml) and in combination with other antifungal agents including Congo Red (60 μg/ml), Calcofluor White (0.3 mg/ml), iprodione (8.6 μg/ml), fludioxonil (0.023 μg/ml), benomyl (0.65 μg/ml), difenoconazole (0.025 μg/ml), and tebuconazole (0.0125 μg/ml). The plates were incubated at 25°C for 4 days.

### Trichothecene Analysis

The total trichothecene produced (deoxynivalenol and 15-acetyl-deoxynivalenol) by *F. graminearum* was measured as described previously (Son et al., 2011a). Fungal strains were grown in minimal media containing 5 mM agmatine (MMA) supplemented with the butanol extract (0, 15, or 30 μg/ml). Seven days later, the cultures were filtered through cheesecloth, and the filtrates were extracted with an ethyl acetate–methanol solution (4:1, v/v). The dehydrated extracts were derivatized with a trimethylsilylating reagent (BSA + TMCS + TMSI, 3:2:3; Supelco, Bellefonte, PA, United States) and analyzed on a Shimadzu QP-5000 gas chromatography mass spectrometer (GC-MS; Shimadzu, Kyoto, Japan). Total trichothecene production was quantified based on the biomass produced by each strain in MMA. The experiment was repeated five times.

### Quantitative Real-Time PCR (qRT-PCR)

Total RNA was extracted from mycelia ground in liquid nitrogen using the Easy-Spin Total RNA Extraction Kit (iNtRON Biotech, Seongnam, South Korea) and cDNA was synthesized using SuperScript III reverse transcriptase (Invitrogen, Carlsbad, CA, United States). qRT-PCRs were performed using SYBR Green Super Mix (Bio-Rad, Hercules, CA, United States) and a 7500 real-time PCR system (Applied Biosystems, Foster City, CA, United States) using the primer pairs for *TRI5* and *TRI6* (Supplementary Table 1). The cyclophilin gene (*CYP1*; FGSG_07439) was used as the reference gene. qRT-PCRs were performed three times with two replicates per run, and the transcript level of each target gene was calculated as described previously (Livak and Schmittgen, 2001).

### Disease Control Efficacy of the JCK-12 Fermentation Broth against FHB on Wheat

Five seeds of ‘Eunpamil’ wheat (*Triticum aestivum* L.) were sown in the nursery soil in vinyl pots (5 cm diameter) and then stored at 4°C for 3 weeks. The seedlings were then grown in the greenhouse for 1 week and followed by transplantation into single plastic pots (20 cm diameter). The plants were grown for roughly 8 weeks in the greenhouse at 25 ± 10°C. Ten spikes were chosen per pot and used for the *in vivo* bioassay. The fermentation broth of JCK-12 was diluted with distilled water by 5- and 20-fold, and Tween-20 was then added to each solution at a concentration of 250 μg/ml as a wetter. Tween-20 solution was used as a negative control. Almuri (Syngenta, Korea), which contains 13% of difenoconazole and 13% of propiconazoles as active ingredients, was used as a positive control at a 2,000-fold dilution. A spore suspension of *F. graminearum* (2 × 10^5^ spores/ml) was sprayed at 1 day after fungicide treatment. The aerial part of each pot was covered with a plastic bag for moisture maintenance. After 3 days, the plastic bag was removed from each pot. After 2 weeks incubation in a greenhouse, both disease incidence (DI, percentage of infected spikes) and severity (DS, percentage of infected spikes among the diseased spikes) of 10 spikes per plot were estimated to yield plot disease severity (FHB index = incidence × severity/100).

A field experiment was conducted using ‘Eunpamil’ at an experimental farm at Chonnam National University (Gwangju, South Korea). Each treatment plot consisted of three 2 m rows with a row spacing of 50 cm. The compound treatments and infection process, same as the greenhouse experiment, were performed, and Almuri (2,000-fold dilution) and Tween-20 solutions were applied as positive and negative controls, respectively. *F. graminearum* spores of were inoculated at 2 × 10^5^ spores/ml using a compressed air sprayer at approximately 2 h before sunset. Both DI and DS for 30 spikes per plot were estimated at 2 weeks after inoculation. Plots were arranged as a randomized complete block design with three replicates per treatment.

### Formulation of a Wettable Powder and Its Disease Control Efficacy against FHB on Wheat

To assess the potential of JCK-12 formulation as the biocontrol agent against FHB, the JCK-12 fermentation broth was dried using a pilot spray dryer (Yoojin Tech. Co., Ltd., South Korea). The dried powder (20 g) was mixed with 15 g of synthetic hydrated silicon dioxide (Rhodia Asia Pvt. Ltd., Singapore), 5 g of sodium dodecyl sulfate (Yoosung Chemical R&T Co., Ltd., South Korea) as a surfactant or wetting agent, 5 g of sodium polynaphthalene formaldehyde (Yoosung Chemical R&T Co., Ltd., South Korea) as a dispersal agent, and 55 g of kaolin. The JCK-12 formulation (JCK-12 WP20) was milled in a blender.

The JCK-12 WP20 was diluted 500-fold with tap water. Tap water was used as a negative control. Almuri (Syngenta), at a 2,000-fold dilution was used as a positive control. The synergistic effect of the JCK-12 formulation and the synthetic antifungal agents was tested using the susceptible wheat ‘Eunpamil’ in the same manner as described in the disease control efficacy experiment with the fermentation broth of JCK-12 against FHB (Lee et al., 2009). Both DI and DS for 30 spikes per plot were estimated at 2 weeks after inoculation. Plots were arranged as a randomized complete block design with three replicates per treatment.

To visualize the effects of JCK-12 WP20 (500-fold dilution), Almuri (4000-fold dilution) and their mixture (500-fold dilution of JCK-12 WP20 with 4000-fold dilution of Almuri) during the infection process of *F. graminearum* in wheat spikes, spore suspensions (10 μl of 1 × 10^5^ spores/ml) harvested from carboxymethyl cellulose (CMC) inoculated with GFP-tagged strains were injected into the center spikelets of the wheat heads at the mid-anthesis after pretreatment of wheat with the JCK-12 formulation and Almuri, each alone and in combination. Six days after inoculation, the wheat spikes infected with GFP-tagged strains were evaluated. Free-hand longitudinal sections of spikes were cut using a clean scalpel and the sectioned spikes were viewed under reflected light and fluorescent light (excitation 470 and emission 525) using a SteREO Lumar V12 microscope (Carl Zeiss, Oberkochen, Germany).

### Statistical Analyses

The inhibitory activity of spore germination and FHB index were subjected to analysis of variance. Means were separated at *P* ≤ 0.05 using a Duncan test (SPSS Statistics, ver. 21, IBM).

## Results

### Screening and Identification of Antifungal Bacteria

**FIGURE 1:**
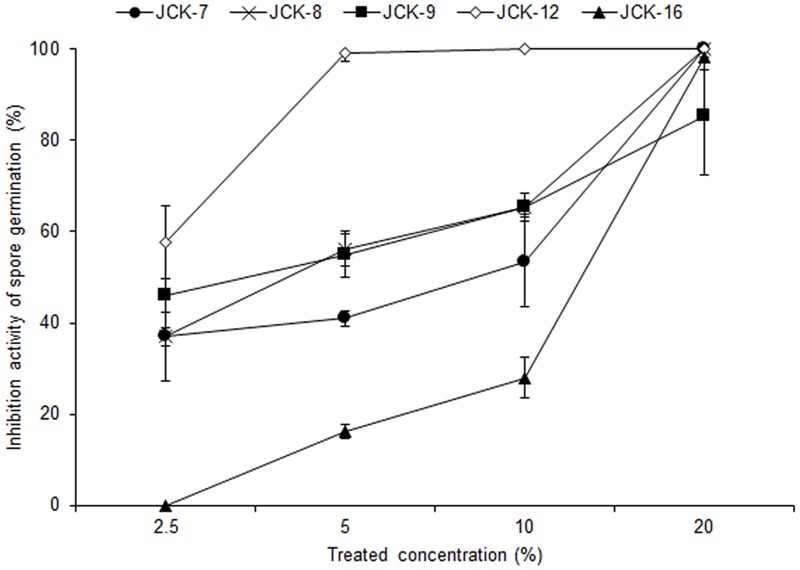
Inhibitory activity of bacterial culture supernatants against *F. graminearum* spore germination.

**FIGURE 2:**
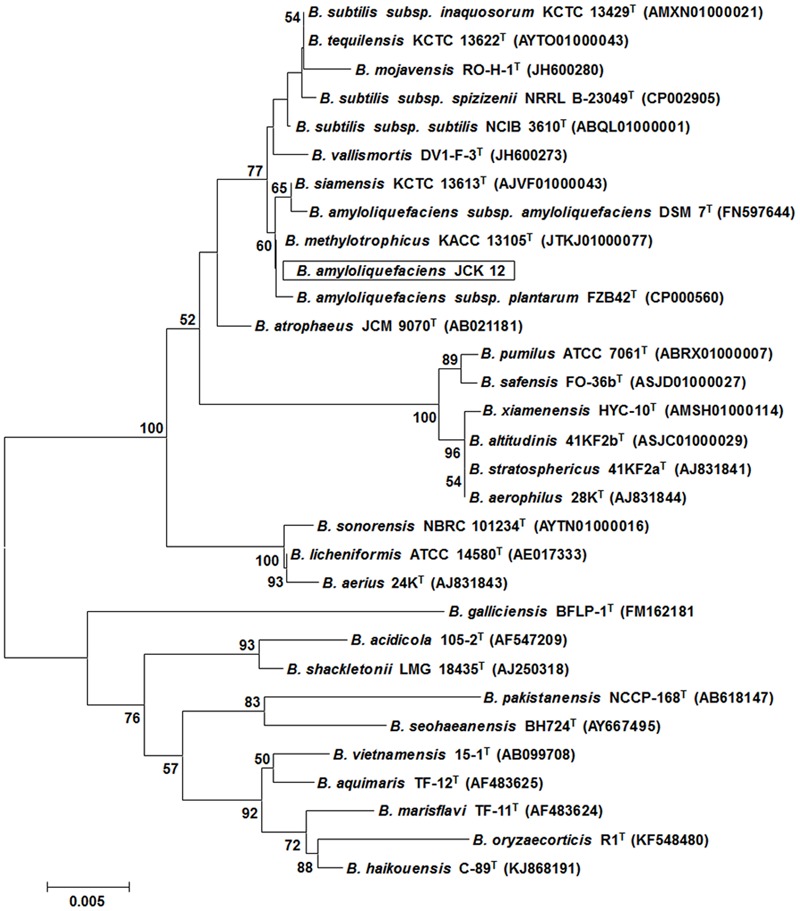
Phylogenetic trees derived from distance analysis of 16S rRNA gene sequences of JCK-12. Sequences were aligned using MEGA 6.0 software. Phylogenetic trees were constructed using the neighbor-joining (NJ) method with bootstrap analysis (1,000 trials). Bars indicate the percentage of sequence divergence.

Five hundred bacterial strains were isolated from the rhizosphere of four different regions in Korea (Daejeon, Gwangju, Jeongeup, and Geoje). Of these 500 rhizospheric bacteria, five strains (JCK-7, JCK-8, JCK-9, JCK-12, and JCK-16) exhibited distinct inhibitory effects on the mycelial growth of *F. graminearum* in a dual culture assay (Supplementary Table 2). Among these, the fermentation culture filtrate of strain JCK-12 showed the strongest inhibition activity against *F. graminearum* spore germination, yielding the minimum inhibitory concentration (MIC) values of 5% (**Figure 1**). Strain JCK-12 was identified as *Bacillus amyloliquefaciens* based on BLASTn analysis and phylogenetic analyses of the amplified 16S rRNA, *gyrA*, and *recA* gene sequences (**Figure 2** and Supplementary Figures 1A,B). The nucleotide sequences of 16S rRNA, *gyrA* and *recA* of strain JCK-12 were deposited in GenBank under accession numbers KT964221, KU963797 and KU963798, respectively.

### Inhibitory Activity of *B. amyloliquefaciens* JCK-12 against Mycelial Growth of Various Plant Pathogenic Fungi

**FIGURE 3:**
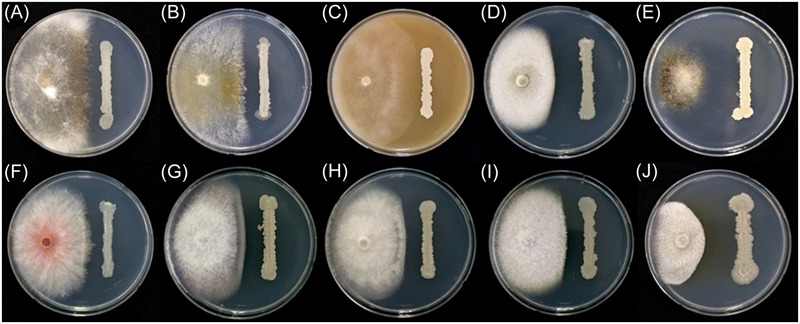
Antifungal activity of strain JCK-12 against various plant pathogen fungi. **(A)** *Rhizoctonia solani*, **(B)** *R. quercus-mongolicae*, **(C)** *P. capsici*, **(D)** *C. coccodes*, **(E)** *B. cinerea*, **(F)** *F. graminearum*, **(G)** *F. oxysporum* f. sp. *niveum*, **(H)** *F. oxysporum* f. sp. *lycopersici*, **(I)** *F. verticillioides*, and **(J)** *M. oryzae* on plates.

In addition to *F. graminearum, B. amyloliquefaciens* JCK-12 inhibited the mycelial growth of all tested plant pathogenic fungi in the dual culture assay (**Figure 3** and Supplementary Table 3). The mycelial growth of *Magnaporthe oryzae* and *Botrytis cinerea* was highly inhibited with distinct inhibition zones. *Colletotrichum coccodes, Raffaelea quercus-mongolicae, Rhizoctonia solani*, and four species of *Fusarium* (*F. graminearum, F. oxysporum* f. sp. *niveum, F. oxysporum* f. sp. *lycopersici*, and *F. verticillioides*) were also sensitive to JCK-12, whereas *Phytophthora capsici*, an oomycete fungus, was relatively insensitive to JCK-12. The results demonstrate that *B. amyloliquefaciens* JCK-12 has a broad spectrum of antifungal activity and could also be used as a biocontrol agent for various plant fungal pathogens used in this analysis.

### Purification of Antifungal Cyclic Lipopeptides

**FIGURE 4:**
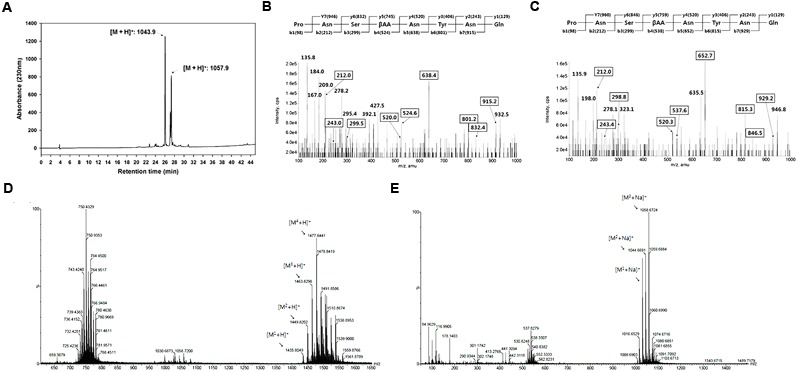
Structural analyses of cyclic lipopeptides produced by *B. amyloliquefaciens* JCK-12. **(A)** HPLC chromatogram of antifungal metabolites, **(B)** LC-MS/MS of iturin A2 ([M + H]^+^: 1,043.9), **(C)** LC-MS/MS of iturin A3 (or A4, A5) ([M + H]^+^: 1,057.9), and **(D)** ESI-MS spectra of F1 and **(E)** F2 isolated from the fermentation broth culture of *B. amyloliquefaciens* JCK-12 by prep TLC. F1 and F2 represent fengycin and surfactin, respectively.

The antifungal active compounds produced by *B. amyloliquefaciens* JCK-12 were purified using a chromatography and bioassay series. The isolated antifungal fraction appeared as a single spot on the TLC plate, but three peaks were observed in the LC-MS/MS analysis (**Figure 4A**). The protonated ions ([M + H]^+^) of the three peaks at 26.15 min (compound 1), 27.99 min (compound 2), and 28.14 min (compound 3) appeared at *m/z* 1043.9, *m/z* 1057.9, and *m/z* 1057.9, respectively. The differences of 14 Da suggest that they are homologous molecules with different lengths of fatty acid chains. In addition, the same protonated ions of compounds 2 and 3 suggested that the two compounds are isomers.LC-MS/MS analysis revealed that compound 1 was iturin A_2_ and that compounds 2 and 3 were one of iturin A_3_, A_4_, and A_5_ (**Figures 4B,C**).

Some *Bacillus* species simultaneously produce other cyclic lipopeptides (CLPs) along with iturin A (Romero et al., 2007; Kim et al., 2010). We further purified two CLPs, fraction 1 and 2, by prep-TLC using the butanol extract of the JCK-12 fermentation broth and analyzed both fractions by HPLC and ESI-MS/MS. The protonated ions ([M + H]^+^) of fraction 1 appeared at *m/z* 1477.8. The protonated ions ([M + Na]^+^) of fraction 2 appeared at *m/z* 1058.7 (**Figures 4D,E**). Fractions 1 and 2 were identified as fengycin and surfactin through ESI-MS/MS analysis. The isolated CLPs were further confirmed using HPLC analysis and comparison with standard chemicals (data not shown). Taken together, these results indicated that *B. amyloliquefaciens* JCK-12 produces three CLPs, iturin A, fengycin, and surfactin.

### Effects of CLPs on *F. graminearum* Conidia Germination

**FIGURE 5:**
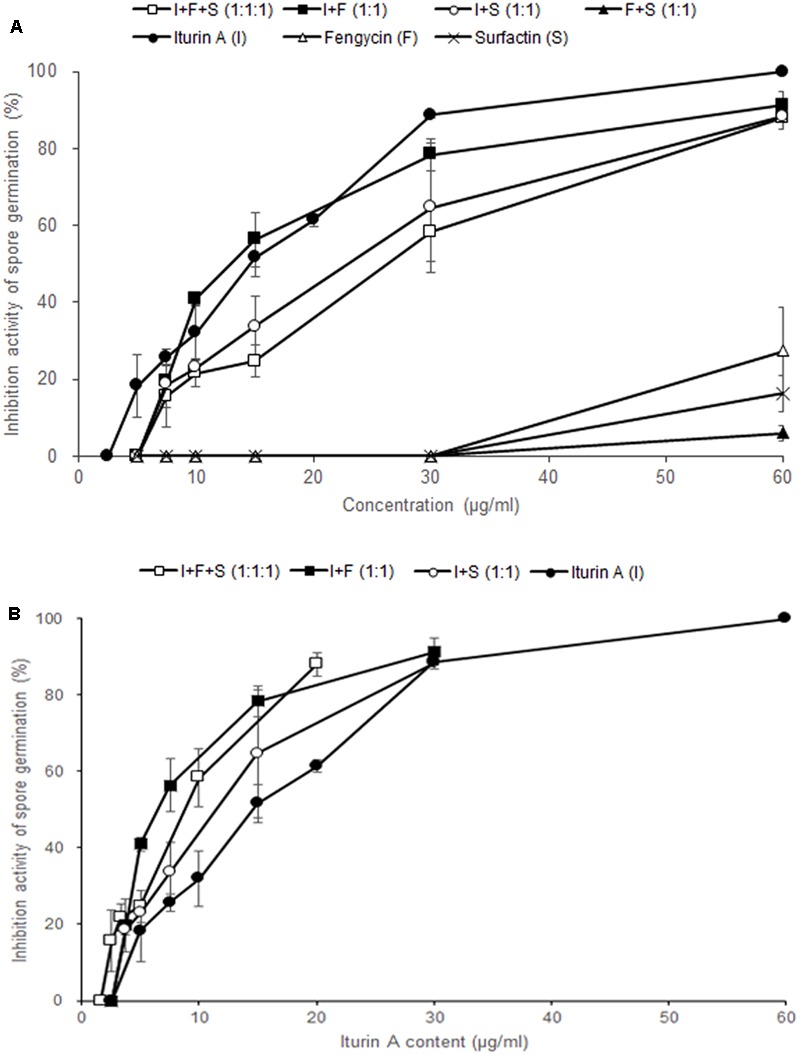
Effect of commercial CLPs alone or in mixture on *F. graminearum* macroconidia germination. **(A)** Based on the total concentration of the three CLPs in solutions, and **(B)** based on the concentration of iturin A alone in the solutions. Error bars represent standard deviation from three replicates.

In order to examine the antifungal effects among the three CLPs on *F. graminearum*, we performed the conidia germination assay with three commercial CLPs in different combinations. At a concentration of 30 μg/ml, the germination inhibitory activities of iturin A, fengycin, surfactin, I + F + S (1:1:1, v/v/v), I + F (1:1, v/v), I + S (1:1, v/v), and F + S (1:1, v/v) on *F. graminearum* conidia were 88.7, 0, 0, 58.3, 78.3, 64.7, and 0%, respectively (**Figure 5A**). Of the three CLPs at 30 μg/ml, only iturin A effectively inhibited conidia germination in *F. graminearum*, whereas fengycin and surfactin had no distinct inhibitory activity at concentrations less than 30 μg/ml. Most mixtures of CLPs had an inhibitory activity on conidia germination at 30 μg/ml except in the treatment F + S (1:1, v/v). These results verified that only iturin A among the three CLPs had effective antifungal activity against *F. graminearum*.

To investigate the synergistic effects of iturin A and other CLPs, we analyzed the inhibitory activity of each treatment according to the content of iturin A (**Figure 5B**). In other words, in I + F (1:1) and I + S (1:1) at 30 μg/ml, the content of iturin A is 15 μg/ml whereas the content of iturin A is 10 μg/ml in I + F + S (1:1:1) at 30 μg/ml. The mixture treatments of I + F (1:1, 78.3%) and I + S (1:1, 64.7%) containing 15 μg/ml of iturin A showed higher inhibitory activity compared to the sole treatment with iturin A (51.7%). Moreover, treatment with the I + F + S (1:1:1, 58.3%) mixture containing 10 μg/ml of iturin A exhibited approximately two-fold increase in the germination inhibitory activity compared with that upon the sole treatment with iturin A (32.0%). These results suggest that co-application of fengycin and surfactin with iturin A enhances the effectiveness of iturin A against *F. graminearum*.

### Effect of the Butanol Extract on Hyphae of *F. graminearum*

Through the analysis of the mycelial growth inhibitory effects of JCK-12 and its butanol extract using the paper disk agar diffusion and pour plate methods, we confirmed that the butanol extract of *B. amyloliquefaciens* JCK-12 containing three CLPs exhibited a strong antifungal activity against *F. graminearum* (Supplementary Figure 2). We used the butanol extract of *B. amyloliquefaciens* JCK-12 as the CLP mixture in the following experiments.

**FIGURE 6:**
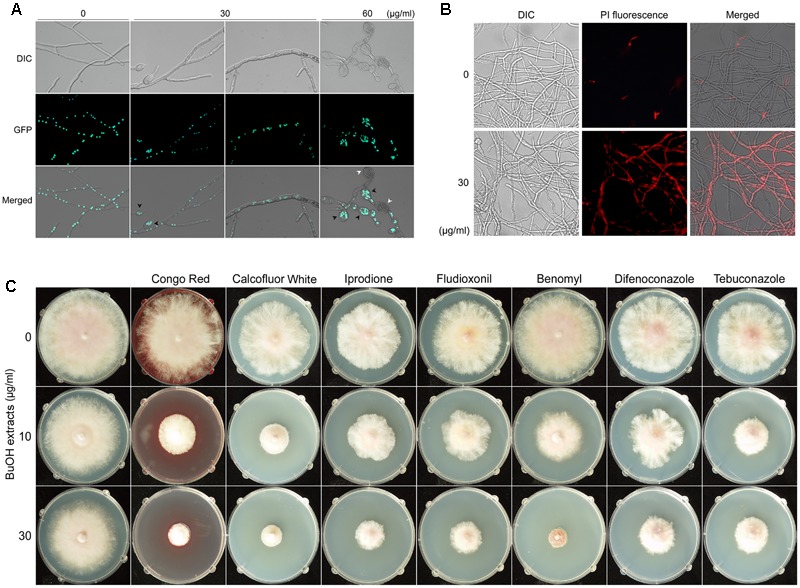
Effects of *B. amyloliquefaciens* JCK-12 on *F. graminearum*. Effects of BuOH extracts on hyphal morphology **(A)**. Green fluorescent dots represent histone H1-green fluorescent protein (GFP). Black arrowheads indicate the balloon-shaped cells and white arrowheads indicate the exploded cells. DIC, differential interference contrast image; GFP, fluorescence microscopy image; Merged, overlays of the DIC and fluorescence microscopy images. The effect of BuOH extracts on cell membrane permeability was examined by propidium iodide (PI) staining **(B)**. Mycelia treated with BuOH extracts (0 or 30 μg/ml) were incubated in 2 μM PI for 20 min. Red fluorescence represents PI-stained hyphae. DIC, differential interference contrast image; PI, fluorescence microscopy image; Merged, overlays of the DIC and fluorescence microscopy images. The synergistic antifungal effect of BuOH extracts and other antifungal compounds on the hyphal growth of *F. graminearum* **(C)**.

Microscopic examination revealed that treatment with the butanol extract affected the hyphal morphology of *F. graminearum* (**Figure 6A**). When the fungus was treated with 30 μg/ml of butanol extract, hyphae were swollen and a few balloon-shaped cells were observed. Upon treatment with a relatively high concentration of the butanol extract (60 μg/ml), fungal growth was severely retarded and many balloon-shaped cells were observed; some of these cells eventually exploded. To visualize the nuclei in fungal cells, a previously generated strain hH1-GFP (Hong et al., 2010) was used. We found that multiple nuclei were present in the balloon-shaped cells compared to those in the untreated control (**Figure 6A**). Similar phenotypic defect was previously reported in deletion mutants of chitin synthase genes in *F. graminearum* (Kim et al., 2009). Moreover, a recent study suggested that iturin A and plipastatin cause damages to the cell walls and plasma membranes of this fungus (Gong et al., 2015).

Based on the previous and current results on hyphal morphology, we hypothesized that CLPs produced by *B. amyloliquefaciens* JCK-12 would damage the cell walls and membrane structures in *F. graminearum*. Since cell membrane damages could increase the cell membrane permeability (Lee and Kim, 2016), we performed propidium iodide (PI) staining to detect the effects of the butanol extract on the fungal cell membrane permeability of *F. graminearum* (**Figure 6B**). PI is a fluorescent probe that is unable to penetrate the cell membrane of healthy cells. However, it can enter the membrane-compromised cells (Cox et al., 2001). We found that the red fluorescence of hyphae treated with the butanol extract was significantly stronger than that of the untreated control. Incubation with 60 μg/ml of the butanol extract showed collapsed hyphae upon visualization of PI staining, indicating extensive cell death (data not shown). Taken together, these results indicated that the CLPs included in the butanol extract markedly increased the cell membrane permeability of *F. graminearum*.

### The Synergistic Antifungal Effect of the Butanol Extract with Antifungal Agents

To determine whether changes in *F. graminearum* cell membrane permeability enhance the antifungal effects of other chemical agents such as Congo Red, Calcoflour White, iprodione, fludioxonil, benomyl, difenoconazole, and tebuconazole, we evaluated the individual and the synergistic antifungal activities of the butanol extract and other antifungal agents on the hyphal growth of *F. graminearum*. Congo Red and Calcofluor White affect fungal cell wall morphogenesis (Roncero and Duran, 1985). Iprodione, fludioxonil, benomyl, difenoconazole, and tebuconazole are used as fungicides that inhibit the germination of fungal spores and the hyphal growth of fungi (Edwards et al., 2001; Gupta et al., 2004; Kojima et al., 2006; Miñambres et al., 2010; Dzhavakhiya et al., 2012). We found a distinct synergistic interaction between the butanol extract and other antifungal agents (**Figure 6C**). Growth inhibition was significantly increased when these antifungal agents were combined with the butanol extract. These results suggest that co-application of the CLPs included in the butanol extract and other antifungal chemicals can inhibit the fungal growth more effectively because of changes in cell membrane permeability. Thus, CLPs produced by *B. amyloliquefaciens* JCK-12 may enhance pathogen sensitivity to fungicides.

### Effects of CLPs on Hyphal Morphology

**FIGURE 7:**
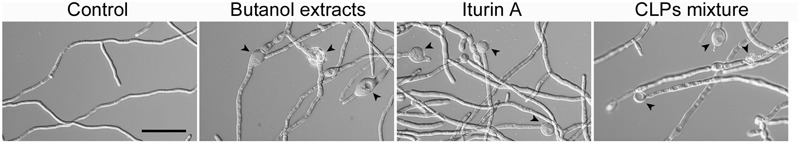
Effect of commercial CLPs on hyphal morphology. Mycelia treated with BuOH extracts (0 or 30 μg/ml), iturin A (15 μg/ml), or the three CLPs mixture (iturin A + fengycin + surfactin = 1.25 + 1.25 + 1.25 μg/ml) were cultured at 200 rpm and 25°C. Black arrowheads indicate the balloon-shaped cells and white arrowheads indicate the exploded cells. Scale bar: 50 μm.

To confirm whether the activity of the butanol extract on *F. graminearum* results from the iturin A. fengycin, and surfactin produced by *B. amyloliquefaciens* JCK-12, we compared the effects of commercial CLPs and the butanol extract on the hyphal morphology of *F. graminearum* (**Figure 7**). When the fungus was treated with 30 μg/ml of the butanol extract, 1.25 μg/ml of the three commercial CLPs, or 15 μg/ml of iturin A, the hyphae were swollen and a few balloon-shaped cells were observed. The effect of the butanol extract on *F. graminearum* hyphal morphology was similar to that of commercial iturin A alone or the mixture of three CLPs. Moreover, the synergistic interaction of the three CLPs mixture enhanced the morphological changes at a low concentration of iturin A, which correspond to the single application of iturin A at a high concentration. Based on these results, we supposed that the CLPs included in the butanol extract could damage the cell wall and alter the membrane structure of *F. graminearum* hyphae, which may subsequently affect membrane permeability.

### *In Vitro* Effect of Butanol Extract on Total Trichothecene Production

**FIGURE 8:**
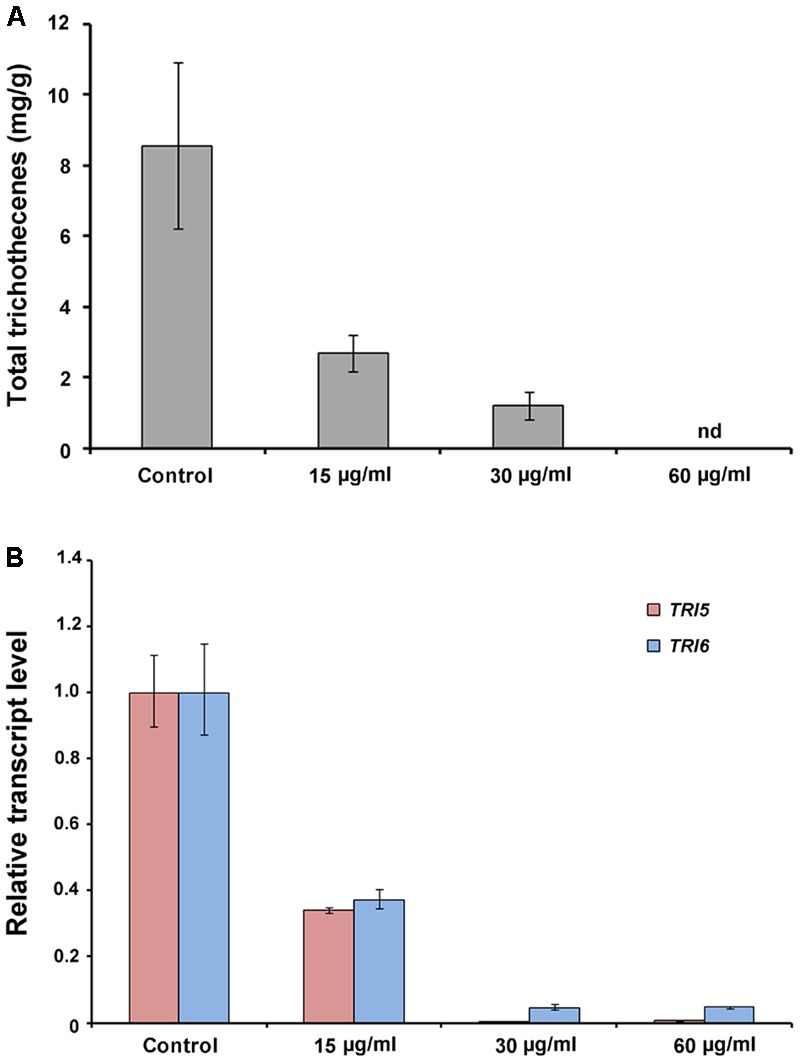
Trichothecene production of *F. graminearum* under CLPs treatment. **(A)** Total trichothecene (DON and 15-acDON) production by *F. graminearum* strains. Fungal strains were grown in liquid minimal media containing 5 mM agmatine (MMA) supplemented with CLPs (0, 15, or 30 μg/ml) for 7 days. Trichothecenes were analyzed by gas chromatography mass spectrometry and were quantified based on the biomass of each strain. **(B)** Transcript levels of *TRI5* and *TRI6* in fungal strains treated with CLPs.

We analyzed the total trichothecene production (deoxynivalenol and 15-acetyl-deoxynivalenol) by *F. graminearum* after treatment with the butanol extract (0, 15, or 30 μg/ml). The accumulation of trichothecenes in the butanol extract-treated cultures was significantly decreased compared with that in the untreated controls (**Figure 8A**). The qRT-PCR results demonstrated that the expression of the trichothecene biosynthetic genes *TRI5* and *TRI6* was also significantly reduced by the butanol extract treatment compared with that in the untreated controls (**Figure 8B**). These results indicate that *B. amyloliquefaciens* JCK-12 plays a crucial role in disrupting trichothecene biosynthesis in *F. graminearum*.

### Effect of JCK-12 Strain on FHB Disease of Wheat

To investigate the efficacy of strain JCK-12 in controlling FHB disease, we estimated the disease incidence, disease severity, and the control value of FHB on wheat under both greenhouse and field conditions after 2 weeks of treatment with the fermentation broth of strain JCK-12 (**Table 1**). In the greenhouse experiment, the fermentation broth of strain JCK-12 showed a dose dependent effect on FHB control with control values of 20.1 and 43.0% at 20- and 5-fold dilution treatments, respectively. The disease incidence and disease severity of the five-fold dilution JCK-12 treatment were comparable to those observed with Almuri. The fermentation broth of JCK-12 also suppressed the development of FHB in the field experiment with control values of 43.8 and 56.3% at 20- and 5-fold dilution treatments, respectively. However, the disease control efficacy of the fermentation broth of JCK-12 at the five-fold dilution was lower than that of Almuri.

**Table 1:** Disease control efficacy of the JCK-12 fermentation broth culture against fusarium head blight caused by *F. graminearum* in wheat under greenhouse and field conditions.

Treatment	Dilution	Greenhouse				Field			
		DI (%)^a^	DS (%)^b^	FHB index^c^	CV (%)^d^	DI (%)	DS (%)	FHB index	CV (%)
JCK-12	×20	52.3ab^e^	29.2b	15.3	20.1	18.0b	4.3c	0.8	43.8
	×5	37.3a	22.1ab	8.2	43.0	14.0b	1.3ab	0.2	56.3
Almuri^f^	×2,000	28.2a	3.4a	1.0	57.0	4.0a	0.4a	0.0	87.5
Untreated control	–	65.5b	37.7b	24.7	–	32.0c	5.0c	1.6	–

*^a^DI, disease incidence. ^b^DS, disease severity. ^c^FHB index = (DI × DS)/100. ^d^CV, control value of disease incidence. ^e^Within columns, means followed by the same lower-case letter are not significantly different (Duncan test, *P* ≤ 0.05). ^f^Almuri was used as a positive control. Dilution value (×2,000) of Almuri represents compatibilizer concentration.*

A wettable powder type formulation of JCK-12 (JCK-12 WP20) was prepared and its disease control efficacy against FHB was then tested under greenhouse and field conditions. In addition, to examine the feasibility of the JCK-12 WP20 formulation as a chemosensitizer to increase the sensitivity of the chemical fungicides used for FHB control in cereals, JCK-12 WP20 was applied in a mixture with the synthetic fungicide, Almuri. Treatment with JCK-12 WP20 was found to reduce both the disease incidence and severity of FHB compared to the untreated control under greenhouse conditions (**Table 2**). However, it did not reduce the development of FHB under field conditions. The mixture of JCK-12 (500-fold dilution) and Almuri (4000-fold dilution) showed the strongest disease control efficacy in both greenhouse and field experiments, followed by 2000- and 4000-fold dilutions of Almuri alone.

**Table 2:** Disease control efficacy of the wettable powder type formulation of JCK-12 against fusarium head blight caused by *F. graminearum* in wheat under greenhouse and field conditions.

Treatment	Dilution	Greenhouse				Field			
		DI (%)^a^	DS (%)^b^	FHB index^c^	CV (%)^d^	DI (%)	DS (%)	FHB index	CV (%)
Almuri	×2,000	26.9a^e^	10.7a	3.3a	94.3	6.2a	39.0ab	2.4a	82.0
	×4,000	61.0bc	20.5ab	14.0ab	79.8	8.8ab	57.4bc	5.6ab	62.6
JCK-12 WP20	×500	76.5bc	54.5b	48.6b	44.0	29.0c	87.5c	25.3c	–
JCK-12 WP20 + Almuri	×500 + ×4,000	31.4ab	7.7a	3.1a	96.4	4.8a	25.1a	2.4a	91.0
Untreated control	–	94.4c	82.0c	78.0c	–	17.5c	76.9c	13.5c	–

*^a^DI, disease incidence. ^b^DS, disease severity. ^c^FHB index = (DI × DS)/100. ^d^CV, control value of disease incidence. ^e^Within columns, means followed by the same lower-case letter are not significantly different (Duncan test, *P* ≤ 0.05). ^f^Almuri was used as a positive control. Dilution value (×2,000) of Almuri represents compatibilizer concentration.*

**FIGURE 9:**
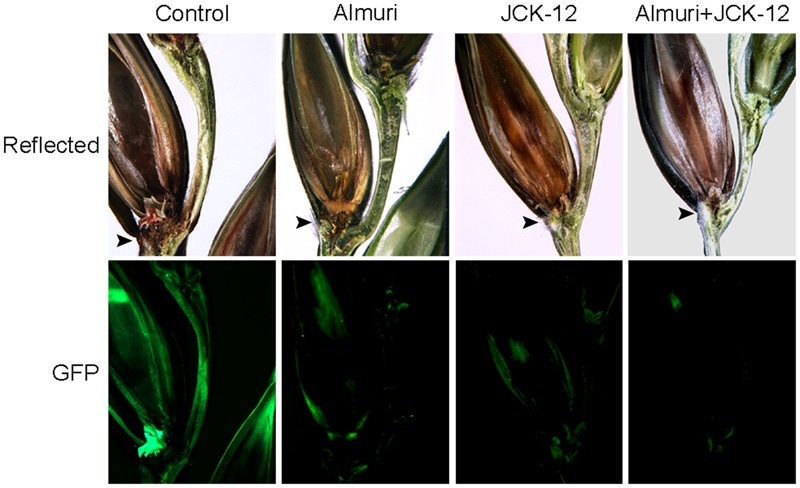
Synergistic control effect of CLPs and the chemical fungicide Almuri. Wheat spikelets pretreated with CLPs and Almuri were inoculated with suspensions of conidia from green fluorescent protein (GFP)-expressing *F. graminearum* strains. The infected wheat heads were dissected at day 7 after inoculation and examined by fluorescence microscopy. Spreading of the GFP signal represents spreading of hyphae from the points of inoculation. Arrowheads mark the inoculated spikelets.

To visualize the spread of mycelia on wheat spikes during infection, the *F. graminearum* strain HK12 constitutively expressing cytosolic GFP was used (Son et al., 2011b). In the wheat spike treated with JCK-12 WP20, Almuri, and their mixture, the fungal hyphae were seen throughout the rachis, but failed to spread to the adjacent spikelet, whereas the fungal hyphae spread through the rachis from the infected to the adjacent spikelet in the untreated wheat spike (**Figure 9**). Especially, the mycelial spread of *F. graminearum* in wheat treated with the combination of JCK-12 WP20 and Almuri was inhibited more than that in any other treatment. These results indicate that JCK-12 WP20 can markedly increase the disease control efficacy of synthetic fungicides when they are applied in a mixture.

## Discussion

Due to the negative effects of agricultural chemicals including environmental pollution, residual chemicals, occurrence of resistant pathogens, and limit period of usage, many studies have been performed to explore a non-hazardous alternative for controlling plant pathogenic fungi and biocontrol agents using various microorganisms (Elkahoui et al., 2012; Ruangwong et al., 2012; Ji et al., 2013). Among them, *Bacillus* species have been regarded as promising candidates for controlling plant pathogens as they have various antagonistic activities and are safe to use (Leifert et al., 1995; Siddiqui et al., 2005; Nagórska et al., 2007; Ji et al., 2013; Zhang et al., 2013; Zhao et al., 2014; Cawoy et al., 2015). In this study, we report that *B. amyloliquefaciens* JCK-12 could be used as a competent biocontrol agent against the cereal head blight fungus, *F. graminearum*. JCK-12 reduced FHB severity in both greenhouse and field conditions. Three CLPs (iturin A, fengycin, and surfactin) produced by JCK-12 cooperatively induced a strong antifungal activity by altering the cell membrane permeability of *F. graminearum*. For practical usage of JCK-12, we verified that the wettable powder type formulation of JCK-12 is an effective biocontrol agent and could be utilized as a chemosensitizer to improve the efficacy of other conventional antifungal chemicals.

*Bacillus amyloliquefaciens* JCK-12 strain produced iturin A, fengycin, and surfactin, which are some of the representative metabolites produced by *Bacillus* species (Ongena and Jacques, 2008). These cyclic lipopeptides mixture exhibited synergistic inhibitory effects on *F. graminearum* spore germination. Among them, it is known that iturin A and fengycin separately have antifungal activity and inhibit the growth of a wide range of plant pathogens, whereas surfactin and iturin A display a synergistic antifungal effect (Romero et al., 2007; Ji et al., 2013; Cawoy et al., 2015). However, our results showed that fengycin alone does not have a powerful antifungal activity against *F. graminearum*, whereas fengycin as well as surfactin increase the antifungal activity of iturin A as synergistic factors. Even the synergistic antifungal activity of fengycin and iturin A is slightly higher than that of surfactin and iturin A on *F. graminearum* spore germination. *B. amyloliquefaciens* JCK-12 strain simultaneously produces three cyclic lipopeptides showing a synergistic antifungal activity against *F. graminearum*, suggesting that strain JCK-12 has the potential to be an excellent biocontrol candidate.

*Fusarium graminearum* accumulates hazardous mycotoxin (DON) on infected grains. However, there is no efficient strategy to prevent DON contamination by late-season infections when chemical fungicides cannot be used (Francl et al., 1999; McMullen et al., 2012). Biological control using *Bacillus* strains has gained importance for the control of mycotoxin accumulation by *Fusarium* species in both pre- and post-harvest crops (Tsitsigiannis et al., 2012). In this study, *B. amyloliquefaciens* JCK-12 was revealed to possess the ability to decrease both fungal growth and DON production. In particular, CLPs successfully inhibited DON production by affecting DON biosynthetic gene expression (**Figure 8**). Conclusively, we demonstrate that biological control using JCK-12 is a promising strategy to prevent mycotoxins as well as FHB itself.

Previous studies have proposed a mechanism for the antifungal action of CLPs using an artificial lipid membrane, demonstrating that iturin A and surfactin penetrate the lipid bilayer of the target cell cytoplasmic membrane and form selective ion-conducting pores, resulting in increased membrane permeability (Sheppard et al., 1991; Maget-Dana and Peypoux, 1994; Carrillo et al., 2003). Furthermore, it is known that treatment with plant hydrolytic enzymes also causes cell wall thinning in the hyphal apex, and subsequently inhibits fungal growth, resulting in an imbalance of turgor pressure and balloon-like swelling of the plasma membrane in fungi (Arlorio et al., 1992). These phenomena are similar to those shown by the cell wall of hyphae treated with Calcofluor White or Congo Red, which disturb growth in yeasts, and in chitinous and cellulosic fungi (Fèvre et al., 1990). In this study, we confirmed that CLPs lead to the formation of balloon-like plasma membrane in *F. graminearum in vivo*. In addition, our results showed that the cell membrane of *F. graminearum* is the essential site of antifungal attack by CLPs and that their antifungal activity may result from altering the fungal cell wall integrity and fungal cell membrane permeability. Moreover, the synergistic effect of the CLPs mixture can significantly enhance cell permeability in fungal hyphae, suggesting that the three cyclic lipopeptides produced by *B. amyloliquefaciens* JCK-12 strain are excellent antifungal sources of biocontrol agents. Through optimization of fermentation conditions for the production of cyclic lipopeptides and appropriate formulation for supporting their stability and control effectiveness, *B. amyloliquefaciens* JCK-12 could be used as a more effective biological agent against FHB.

The treatment of *B. amyloliquefaciens* JCK-12 extracts or formulation and chemical fungicides in combination caused cell wall damage in the hyphae of *F. graminearum* and subsequent increased sensitivity of *F. graminearum* to fungicides, indicating that *B. amyloliquefaciens* JCK-12 can be used as candidates for chemosensitizers. Antifungal chemosensitization is a novel antifungal intervention strategy to improve the efficacy of conventional agents against fungal pathogens (Niimi et al., 2004; Kim et al., 2008). Although a single application of the *B. amyloliquefaciens* JCK-12 formulation is not a complete alternative for synthetic chemicals to control FHB, *B. amyloliquefaciens* JCK-12 is a good biocontrol candidate that produces potent chemosensitizers. Therefore, our results are progressive and noteworthy in that *B. amyloliquefaciens* JCK-12 can actually increase antifungal activity under field conditions and reduce the input of chemical fungicides as supplements or chemosensitizers in a system of integrated plant disease management with no safety problems to humans or the environment.

In addition, use of this biological agent enables extension of its application period past the flowering stage when synthetic fungicides can no longer be applied. A biological control system using *B. amyloliquefaciens* JCK-12 may show great potential for reducing mycotoxin accumulation. This finding shows that *B. amyloliquefaciens* JCK-12 has promising potential for the development of effective and eco-friendly chemosensitizers that could be used in combination with chemical fungicides, to decrease the incidence of resistance development in pathogens, to reduce environmental damage by lowering the effective dosage levels of chemical fungicides, and to enhance the efficacy of antifungal agents. Our study has important practical implications in that FHB management was performed using a formulation of *B. amyloliquefaciens* JCK-12 under the field condition and we identified *B. amyloliquefaciens* JCK-12 as an potential candidate for the chemosensitizer to be used with agrochemicals based on the results of the field trials.

## Author Contributions

KK, YL, AP, and J-CK conceived this study. KK, YL, AH, J-IK, AP, NY, HP, CL, TL, and J-CK performed the experiments. KK, AP, HS, GC, Y-WL, and J-CK analyzed data. AP, KK, YL, HS, Y-WL, and J-CK wrote the manuscript. All authors approved the manuscript.

## Conflict of Interest Statement

The authors declare that the research was conducted in the absence of any commercial or financial relationships that could be construed as a potential conflict of interest.
