# Supplementary material for: Chemosensitization of Fusarium graminearum to Chemical Fungicides Using Cyclic Lipopeptides Produced by Bacillus amyloliquefaciens Strain JCK-12
Source: Front Plant Sci. 2017 Nov 27;8:2010. doi: 10.3389/fpls.2017.02010 (PMC5711811; doi:10.3389/fpls.2017.02010)
Supplement: Supplementary file 1 [file Table_1.DOCX]

**Supplementary Table 1** **|** Primers used in this study.

| **Primer** | **Sequence (5' → 3')** | **Description** |
| --- | --- | --- |
| 16S-9F | GAGTTTGATCCTGGCTCAG | For amplification of 16S rRNA |
| 16S-1492R | GGTTACCTTGTTACGACTT |  |
| gyrA-F | CAGTCAGGAAATGCGTACGTCCTT | For amplification of *gyrA* |
| gyrA-R | CAAGGTAATGCTCCAGGCATTGCT |  |
| recA-F | GATCGTCARGCAGSCYTWGAT | For amplification of *recA* |
| recA-R | TTWCCRACCATAACSCCRAC |  |
| TRI5-RT-F | GCCATTTTGGACCTTTCTGCTCATT | For realtime-PCR of *TRI5* |
| TRI5-RT-R | GCCATAGAGAAGCCCCAACACAAT |  |
| TRI6-RT-F | GGCAACCATTCAAGCGCTTTTTCT | For realtime-PCR of *TRI6* |
| TRI6-RT-R | CACCCTGCTAAAGACCCTCAGACATT |  |
| CYP1-RT-F | TCAAGCTCAAGCACACCAAGAAGG | For realtime-PCR of *CYP1* |
| CYP1-RT-R | GGTCCGCCGCTCCAGTCT |  |
