# Supplementary material for: Chemosensitization of Fusarium graminearum to Chemical Fungicides Using Cyclic Lipopeptides Produced by Bacillus amyloliquefaciens Strain JCK-12
Source: Front Plant Sci. 2017 Nov 27;8:2010. doi: 10.3389/fpls.2017.02010 (PMC5711811; doi:10.3389/fpls.2017.02010)
Supplement: Supplementary file 2 [file Table_2.DOCX]

**Supplementary Table 2 |** Antifungal activities of bacterial strains against *F. graminearum* growth in a dual culture assay

| Strain | Inhibition activity | Strain | Inhibition activity | Strain | Inhibition activity |
| --- | --- | --- | --- | --- | --- |
| JCK-1 | - | JCK-168 | - | JCK-335 | ** |
| JCK-2 | - | JCK-169 | - | JCK-336 | * |
| JCK-3 | - | JCK-170 | - | JCK-337 | - |
| JCK-4 | - | JCK-171 | * | JCK-338 | - |
| JCK-5 | - | JCK-172 | - | JCK-339 | - |
| JCK-6 | - | JCK-173 | - | JCK-340 | * |
| JCK-7 | **** | JCK-174 | - | JCK-341 | * |
| JCK-8 | **** | JCK-175 | - | JCK-342 | - |
| JCK-9 | **** | JCK-176 | - | JCK-343 | - |
| JCK-10 | - | JCK-177 | *** | JCK-344 | *** |
| JCK-11 | - | JCK-178 | * | JCK-345 | - |
| JCK-12 | **** | JCK-179 | - | JCK-346 | - |
| JCK-13 | ** | JCK-180 | - | JCK-347 | - |
| JCK-14 | - | JCK-181 | - | JCK-348 | - |
| JCK-15 | - | JCK-182 | - | JCK-349 | - |
| JCK-16 | **** | JCK-183 | - | JCK-350 | * |
| JCK-17 | - | JCK-184 | - | JCK-351 | - |
| JCK-18 | - | JCK-185 | - | JCK-352 | - |
| JCK-19 | ** | JCK-186 | - | JCK-353 | *** |
| JCK-20 | * | JCK-187 | - | JCK-354 | - |
| JCK-21 | ** | JCK-188 | - | JCK-355 | * |
| JCK-22 | ** | JCK-189 | - | JCK-356 | - |
| JCK-23 | *** | JCK-190 | - | JCK-357 | - |
| JCK-24 | ** | JCK-191 | - | JCK-358 | - |
| JCK-25 | ** | JCK-192 | * | JCK-359 | - |
| JCK-26 | *** | JCK-193 | *** | JCK-360 | - |
| JCK-27 | *** | JCK-194 | *** | JCK-361 | - |
| JCK-28 | ** | JCK-195 | - | JCK-362 | *** |
| JCK-29 | *** | JCK-196 | - | JCK-363 | - |
| JCK-30 | *** | JCK-197 | - | JCK-364 | - |
| JCK-31 | ** | JCK-198 | - | JCK-365 | * |
| JCK-32 | *** | JCK-199 | - | JCK-366 | - |
| JCK-33 | ** | JCK-200 | - | JCK-367 | - |
| JCK-34 | ** | JCK-201 | - | JCK-368 | - |
| JCK-35 | *** | JCK-202 | *** | JCK-369 | - |
| JCK-36 | ** | JCK-203 | *** | JCK-370 | - |
| JCK-37 | *** | JCK-204 | - | JCK-371 | - |
| JCK-38 | *** | JCK-205 | ** | JCK-372 | - |
| JCK-39 | ** | JCK-206 | - | JCK-373 | * |
| JCK-40 | *** | JCK-207 | - | JCK-374 | * |
| JCK-41 | - | JCK-208 | *** | JCK-375 | - |
| JCK-42 | ** | JCK-209 | - | JCK-376 | * |
| JCK-43 | *** | JCK-210 | ** | JCK-377 | - |
| JCK-44 | *** | JCK-211 | *** | JCK-378 | * |
| JCK-45 | - | JCK-212 | - | JCK-379 | * |
| JCK-46 | *** | JCK-213 | - | JCK-380 | - |
| JCK-47 | *** | JCK-214 | - | JCK-381 | * |
| JCK-48 | - | JCK-215 | - | JCK-382 | - |
| JCK-49 | *** | JCK-216 | - | JCK-383 | - |
| JCK-50 | * | JCK-217 | - | JCK-384 | - |
| JCK-51 | *** | JCK-218 | - | JCK-385 | * |
| JCK-52 | - | JCK-219 | *** | JCK-386 | - |
| JCK-53 | *** | JCK-220 | - | JCK-387 | - |
| JCK-54 | *** | JCK-221 | *** | JCK-388 | - |
| JCK-55 | *** | JCK-222 | * | JCK-389 | - |
| JCK-56 | *** | JCK-223 | - | JCK-390 | - |
| JCK-57 | ** | JCK-224 | - | JCK-391 | - |
| JCK-58 | - | JCK-225 | ** | JCK-392 | - |
| JCK-59 | *** | JCK-226 | - | JCK-393 | - |
| JCK-60 | *** | JCK-227 | - | JCK-394 | - |
| JCK-61 | *** | JCK-228 | - | JCK-395 | - |
| JCK-62 | *** | JCK-229 | - | JCK-396 | *** |
| JCK-63 | *** | JCK-230 | *** | JCK-397 | - |
| JCK-64 | *** | JCK-231 | - | JCK-398 | - |
| JCK-65 | *** | JCK-232 | *** | JCK-399 | *** |
| JCK-66 | *** | JCK-233 | ** | JCK-400 | - |
| JCK-67 | ** | JCK-234 | - | JCK-401 | - |
| JCK-68 | - | JCK-235 | - | JCK-402 | - |
| JCK-69 | - | JCK-236 | - | JCK-403 | - |
| JCK-70 | ** | JCK-237 | - | JCK-404 | - |
| JCK-71 | *** | JCK-238 | - | JCK-405 | - |
| JCK-72 | *** | JCK-239 | - | JCK-406 | - |
| JCK-73 | - | JCK-240 | - | JCK-407 | - |
| JCK-74 | - | JCK-241 | - | JCK-408 | - |
| JCK-75 | *** | JCK-242 | *** | JCK-409 | - |
| JCK-76 | - | JCK-243 | - | JCK-410 | - |
| JCK-77 | - | JCK-244 | - | JCK-411 | - |
| JCK-78 | - | JCK-245 | - | JCK-412 | - |
| JCK-79 | - | JCK-246 | - | JCK-413 | - |
| JCK-80 | * | JCK-247 | - | JCK-414 | - |
| JCK-81 | - | JCK-248 | - | JCK-415 | - |
| JCK-82 | - | JCK-249 | - | JCK-416 | - |
| JCK-83 | *** | JCK-250 | - | JCK-417 | - |
| JCK-84 | - | JCK-251 | - | JCK-418 | - |
| JCK-85 | - | JCK-252 | - | JCK-419 | - |
| JCK-86 | - | JCK-253 | - | JCK-420 | - |
| JCK-87 | - | JCK-254 | - | JCK-421 | - |
| JCK-88 | - | JCK-255 | - | JCK-422 | - |
| JCK-89 | - | JCK-256 | - | JCK-423 | - |
| JCK-90 | - | JCK-257 | - | JCK-424 | - |
| JCK-91 | - | JCK-258 | - | JCK-425 | - |
| JCK-92 | - | JCK-259 | - | JCK-426 | - |
| JCK-93 | - | JCK-260 | - | JCK-427 | - |
| JCK-94 | * | JCK-261 | - | JCK-428 | - |
| JCK-95 | ** | JCK-262 | - | JCK-429 | - |
| JCK-96 | - | JCK-263 | * | JCK-430 | - |
| JCK-97 | ** | JCK-264 | - | JCK-431 | - |
| JCK-98 | *** | JCK-265 | - | JCK-432 | ** |
| JCK-99 | - | JCK-266 | - | JCK-433 | - |
| JCK-100 | *** | JCK-267 | - | JCK-434 | - |
| JCK-101 | ** | JCK-268 | - | JCK-435 | - |
| JCK-102 | *** | JCK-269 | - | JCK-436 | - |
| JCK-103 | *** | JCK-270 | - | JCK-437 | - |
| JCK-104 | *** | JCK-271 | - | JCK-438 | - |
| JCK-105 | *** | JCK-272 | *** | JCK-439 | - |
| JCK-106 | ** | JCK-273 | ** | JCK-440 | - |
| JCK-107 | *** | JCK-274 | - | JCK-441 | - |
| JCK-108 | *** | JCK-275 | - | JCK-442 | - |
| JCK-109 | - | JCK-276 | *** | JCK-443 | - |
| JCK-110 | *** | JCK-277 | * | JCK-444 | - |
| JCK-111 | *** | JCK-278 | - | JCK-445 | - |
| JCK-112 | *** | JCK-279 | * | JCK-446 | - |
| JCK-113 | - | JCK-280 | - | JCK-447 | - |
| JCK-114 | *** | JCK-281 | - | JCK-448 | - |
| JCK-115 | *** | JCK-282 | - | JCK-449 | - |
| JCK-116 | ** | JCK-283 | - | JCK-450 | - |
| JCK-117 | *** | JCK-284 | - | JCK-451 | - |
| JCK-118 | - | JCK-285 | - | JCK-452 | - |
| JCK-119 | - | JCK-286 | - | JCK-453 | - |
| JCK-120 | - | JCK-287 | - | JCK-454 | - |
| JCK-121 | - | JCK-288 | - | JCK-455 | - |
| JCK-122 | - | JCK-289 | ** | JCK-456 | - |
| JCK-123 | - | JCK-290 | *** | JCK-457 | - |
| JCK-124 | *** | JCK-291 | ** | JCK-458 | - |
| JCK-125 | - | JCK-292 | - | JCK-459 | - |
| JCK-126 | - | JCK-293 | - | JCK-460 | - |
| JCK-127 | - | JCK-294 | - | JCK-461 | - |
| JCK-128 | - | JCK-295 | - | JCK-462 | - |
| JCK-129 | - | JCK-296 | - | JCK-463 | - |
| JCK-130 | - | JCK-297 | - | JCK-464 | - |
| JCK-131 | - | JCK-298 | - | JCK-465 | - |
| JCK-132 | - | JCK-299 | - | JCK-466 | - |
| JCK-133 | - | JCK-300 | - | JCK-467 | - |
| JCK-134 | - | JCK-301 | - | JCK-468 | - |
| JCK-135 | - | JCK-302 | - | JCK-469 | - |
| JCK-136 | *** | JCK-303 | - | JCK-470 | - |
| JCK-137 | - | JCK-304 | - | JCK-471 | - |
| JCK-138 | ** | JCK-305 | - | JCK-472 | - |
| JCK-139 | - | JCK-306 | - | JCK-473 | * |
| JCK-140 | *** | JCK-307 | ** | JCK-474 | - |
| JCK-141 | * | JCK-308 | - | JCK-475 | - |
| JCK-142 | *** | JCK-309 | - | JCK-476 | - |
| JCK-143 | - | JCK-310 | - | JCK-477 | - |
| JCK-144 | *** | JCK-311 | * | JCK-478 | - |
| JCK-145 | *** | JCK-312 | - | JCK-479 | - |
| JCK-146 | *** | JCK-313 | ** | JCK-480 | - |
| JCK-147 | ** | JCK-314 | ** | JCK-481 | - |
| JCK-148 | *** | JCK-315 | - | JCK-482 | - |
| JCK-149 | - | JCK-316 | - | JCK-483 | - |
| JCK-150 | - | JCK-317 | - | JCK-484 | - |
| JCK-151 | - | JCK-318 | - | JCK-485 | - |
| JCK-152 | * | JCK-319 | - | JCK-486 | - |
| JCK-153 | - | JCK-320 | - | JCK-487 | - |
| JCK-154 | - | JCK-321 | * | JCK-488 | - |
| JCK-155 | - | JCK-322 | - | JCK-489 | - |
| JCK-156 | - | JCK-323 | - | JCK-490 | ** |
| JCK-157 | - | JCK-324 | - | JCK-491 | - |
| JCK-158 | - | JCK-325 | * | JCK-492 | - |
| JCK-159 | * | JCK-326 | * | JCK-493 | - |
| JCK-160 | - | JCK-327 | - | JCK-494 | - |
| JCK-161 | - | JCK-328 | - | JCK-495 | * |
| JCK-162 | - | JCK-329 | - | JCK-496 | - |
| JCK-163 | - | JCK-330 | - | JCK-497 | - |
| JCK-164 | ** | JCK-331 | - | JCK-498 | ** |
| JCK-165 | - | JCK-332 | - | JCK-499 | - |
| JCK-166 | - | JCK-333 | - | JCK-500 | - |
| JCK-167 | - | JCK-334 | - |  |  |

-, no visible inhibition; *, no fungal growth on 0.1–3% of the plate area/bacterial streak; **, no fungal growth on 3–8% of the plate area/bacterial streak; ***, no fungal growth on > 8% of the plate area/bacterial streak. All data were obtained from three replicates.
