# Supplementary material for: Chemosensitization of Fusarium graminearum to Chemical Fungicides Using Cyclic Lipopeptides Produced by Bacillus amyloliquefaciens Strain JCK-12
Source: Front Plant Sci. 2017 Nov 27;8:2010. doi: 10.3389/fpls.2017.02010 (PMC5711811; doi:10.3389/fpls.2017.02010)
Supplement: Supplementary file 3 [file Table_3.DOCX]

**Supplementary Table 3 |** *In vitro* antifungal activity of JCK-12 against various plant pathogenic fungi.

| Plant pathogenic fungi | Inhibition zone (mm) |
| --- | --- |
| *Botrytis cinerea* | 24 |
| *Collectotrichum coccodes* | 19 |
| *Fusarium graminearum* | 12 |
| *F. oxysporum* f. sp*. niveum* | 14 |
| *F. oxysporum* f. sp*. lycopersici* | 14 |
| *F. verticillioides* | 14 |
| *Magnaporthe oryzae* | 25 |
| *Phytophthora capsici* | 9 |
| *Rhizoctonia solani* | 14 |
| *Raffaelea quercus-mongolicae* | 15 |
