# Supplementary material for: Chemosensitization of Fusarium graminearum to Chemical Fungicides Using Cyclic Lipopeptides Produced by Bacillus amyloliquefaciens Strain JCK-12
Source: Front Plant Sci. 2017 Nov 27;8:2010. doi: 10.3389/fpls.2017.02010 (PMC5711811; doi:10.3389/fpls.2017.02010)
Supplement: Supplementary file 4 [file Image_1.PDF]

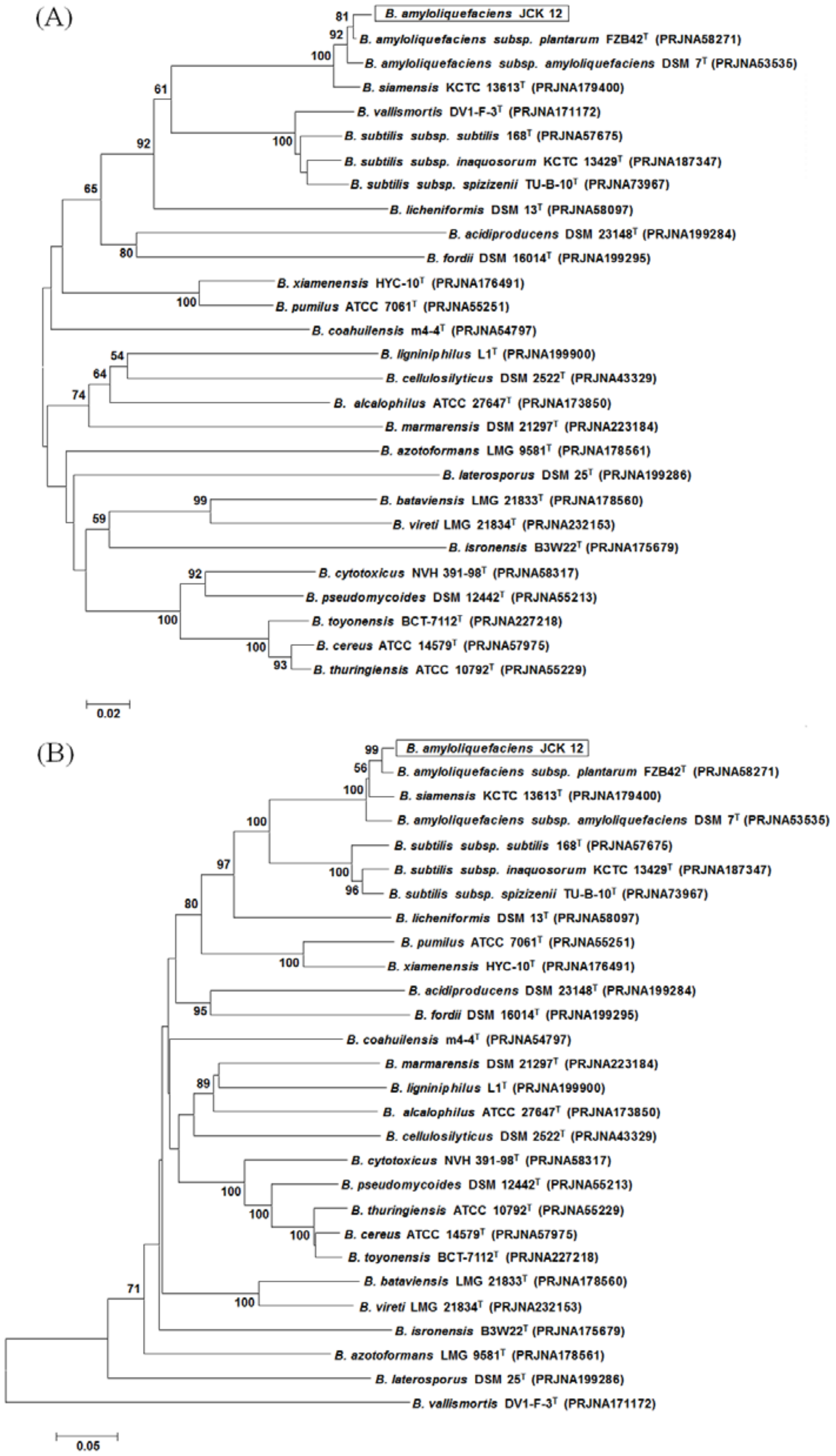

Supplementary Figure 1 | Phylogenetic trees derived from distance analysis of DNA Gyrase A subunit (A) and Rec A protein (B) gene sequences of JCK-12. Sequences were aligned using MEGA 6.0 software. Neighbor-joining (NJ) method phylogenetic tree construction and bootstrap analysis (1,000 trials) were carried out. Bars indicate the percentage of sequence divergence.
