# Supplementary material for: Chemosensitization of Fusarium graminearum to Chemical Fungicides Using Cyclic Lipopeptides Produced by Bacillus amyloliquefaciens Strain JCK-12
Source: Front Plant Sci. 2017 Nov 27;8:2010. doi: 10.3389/fpls.2017.02010 (PMC5711811; doi:10.3389/fpls.2017.02010)
Supplement: Supplementary file 5 [file Image_2.PDF]

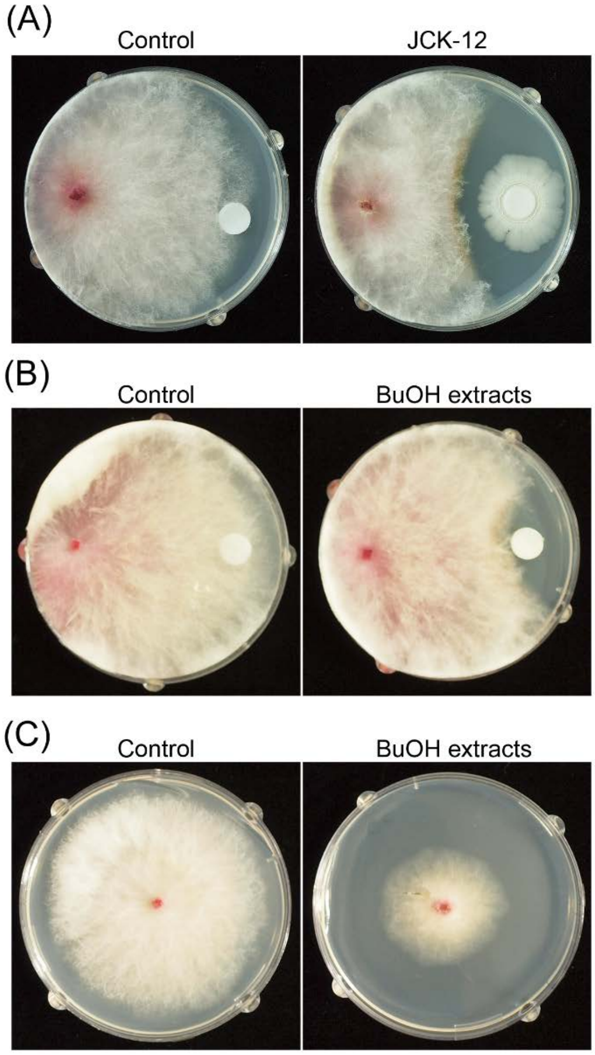

Supplementary Figure 2 | Antifungal activity of JCK-12 and its butanol extract. The antifungal activities of JCK-12 and its butanol (BuOH) extracts on the radial growth of *F. graminearum* were tested using a dual assay (A, B) and the pour plate method (C).
